# An autopsy case of primary extranodal NK/T cell lymphoma (extranodal NK/T-cell lymphoma) of the bile duct

**DOI:** 10.1007/s12328-018-00931-1

**Published:** 2019-01-02

**Authors:** Hiroyuki Ito, Shin-ichiro Hiraiwa, Tomoko Sugiyama, Takuma Tajiri, Yoko Yamaji, Motoki Kaneko, Shingo Tsuda, Hitoshi Ichikawa, Junko Nagata, Seiichiro Kojima, Shinji Takashimizu, Takayuki Shirai, Norihito Watanabe

**Affiliations:** 1 Department of Gastroenterology, Tokai University Hachioji Hospital, Tokyo, Japan; 2 Department of Pathology, Tokai University Hachioji Hospital, Tokyo, Japan

**Keywords:** Extranodal NK/T-cell lymphoma, Bile duct, Prognosis, Digestive organ

## Abstract

We reported the case of a 50-year-old man diagnosed with extrinsic NK/T-cell lymphoma. He was initially diagnosed with locally advanced unresected pancreatic duct carcinoma and was treated with combination chemotherapy using gemcitabine and nabpaclitaxel. One month after treatment, he developed bleeding. Upper gastrointestinal endoscopy showed a deep ulcer lesion from the duodenal bulb to the inner wall of the descending section that was not observed before treatment. Coil embolization was performed, but the necrotic area widened after treatment; the patient died of disseminated intravascular coagulation after 1 week. Autopsy showed a soft white-tone lesion that extended from the ulcer wall to the gallbladder wall and around the intrahepatic bile duct. Lesions were also found in the spleen, lungs, kidney, and bone marrow, and immunohistochemistry confirmed extrinsic NK/T-cell lymphoma (extranodal NK/T-cell lymphoma, nasal type). In conclusion, histological diagnosis of NK/T-cell lymphoma is difficult at an early stage, and the clinical course often shows rapid tumor progression, particularly bleeding in the digestive organs or widespread perforation and penetration. NK/T-cell lymphoma should be ruled out in patients with bile duct and pancreatic tumors in whom tissue diagnosis via biopsy cannot be performed.

## Introduction

Malignant lymphoma of the digestive organs can be common, but malignant lymphomas of the NK/T-cell type are extremely rare, particularly those that involve the pancreaticobiliary system. Only a few cases of NK/T-cell type lymphoma have been reported worldwide. Extranodal NK/T-cell lymphoma is difficult to diagnose via conventional hematoxylin–eosin (HE) staining. It is characterized by a clinical course that shows rapid tumor progression, particularly in the gastrointestinal tract, with bleeding and perforation/penetration as complications; this results in sudden patient deterioration. Herein, we report a case of extranodal NK/T-cell lymphoma of the bile duct that was diagnosed during autopsy.

## Case report

A 50-year-old man consulted a primary doctor for jaundice that had lasted several weeks. Imaging showed a pancreatic head tumor and bile duct dilation; thus, he was referred to our hospital.

**Fig. 1:**
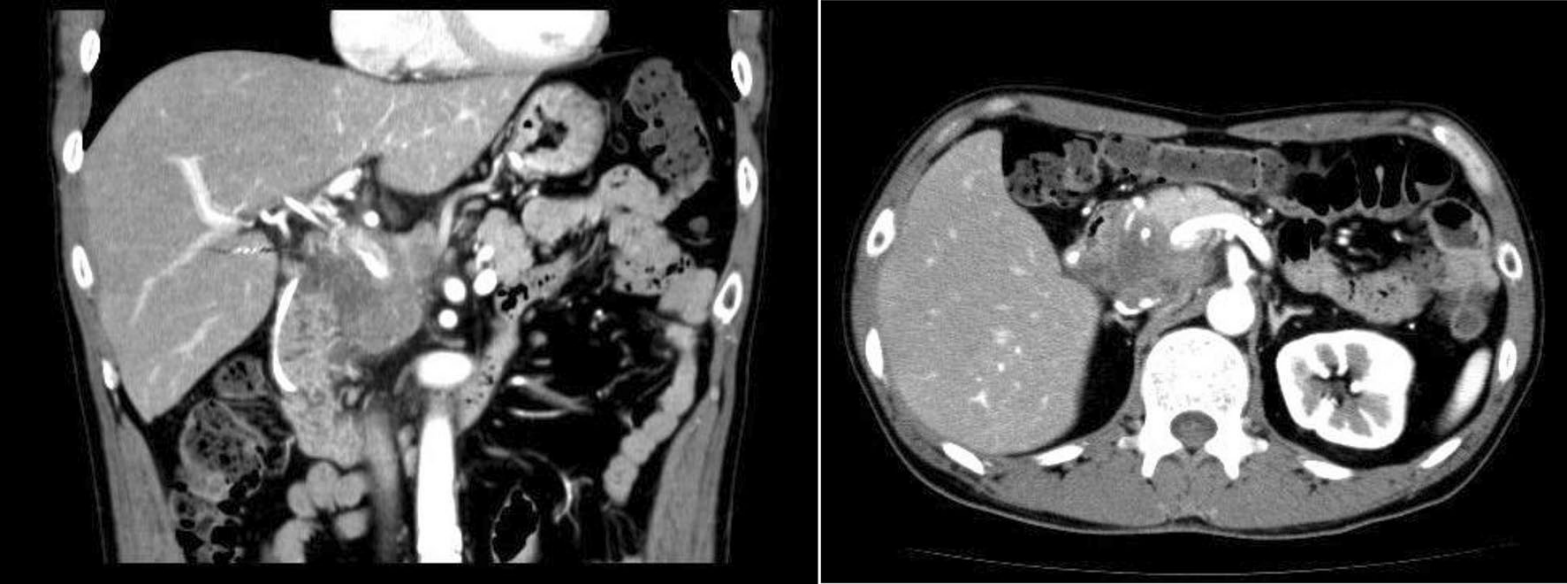
Contrast-enhanced abdominal CT shows tumor involvement in the portal vein and inferior vena cava and soft irregular tissues surrounding the celiac artery

Jaundice was noted on examination, but no other symptoms, such as fever, abdominal pain, and nasal cavity and pharyngeal lesions, were noted. The following blood test results indicated obstructive jaundice: white blood cell count, 6700/µl; hemoglobin level, 12.0 g/dl; platelet count, 393,000/µl; glutamic oxaloacetic transaminase level, 61 IU/l; glutamic pyruvic transaminase level, 114 IU/l; lactate dehydrogenase level, 260 IU/l; total bilirubin, 6.3 mg/dl; direct bilirubin, 4.3 mg/dl; international normalized ratio, 1.05 (prothrombin time 89%); and C-reactive protein level, 0.09 mg/dl. His tumor markers were normal (carcinoembryonic antigen, 4.8 ng/ml; carbohydrate antigen 19–9, 1.0 U/ml; DUPAN-2, 12.0 U/ml; and SPan-1, 39.3 U/ml), but soluble interleukin 2-receptor levels were greatly increased (2770 U/ml). Abdominal contrast-enhanced computed tomography (CT) revealed a tumor at the head of the pancreas that invaded the portal vein, inferior vena cava, and celiac artery (Fig. 1). The mass also caused pancreatic bile duct stenosis. Moreover, the peripheral bile duct was dilated, but not the main pancreatic duct. Positron emission tomography-CT showed enhanced fluorodeoxyglucose uptake in the lesion area and showed no lesions in the head and neck. Moreover, we confirmed that the tumor confined to the head of the pancreas. Endoscopic ultrasound-guided fine needle aspiration of the pancreatic tumor tissue was performed twice using a 25G needle, but there was no evidence of any lymphocytes that suggested lymphoma. Endoscopic retrograde cholangiopancreatography for pancreatic ductal scrub cytology, bile duct tissue, biliary cytology, and pancreatic juice cytology showed no malignancy.

Although a histological diagnosis was not established and the tumor markers were normal, we judged that the possibility of lymphoma was low; therefore, gemcitabine–nabpaclitaxel combination therapy was started after a diagnosis of locally advanced unresectable pancreatic duct cancer.

**Fig. 2:**
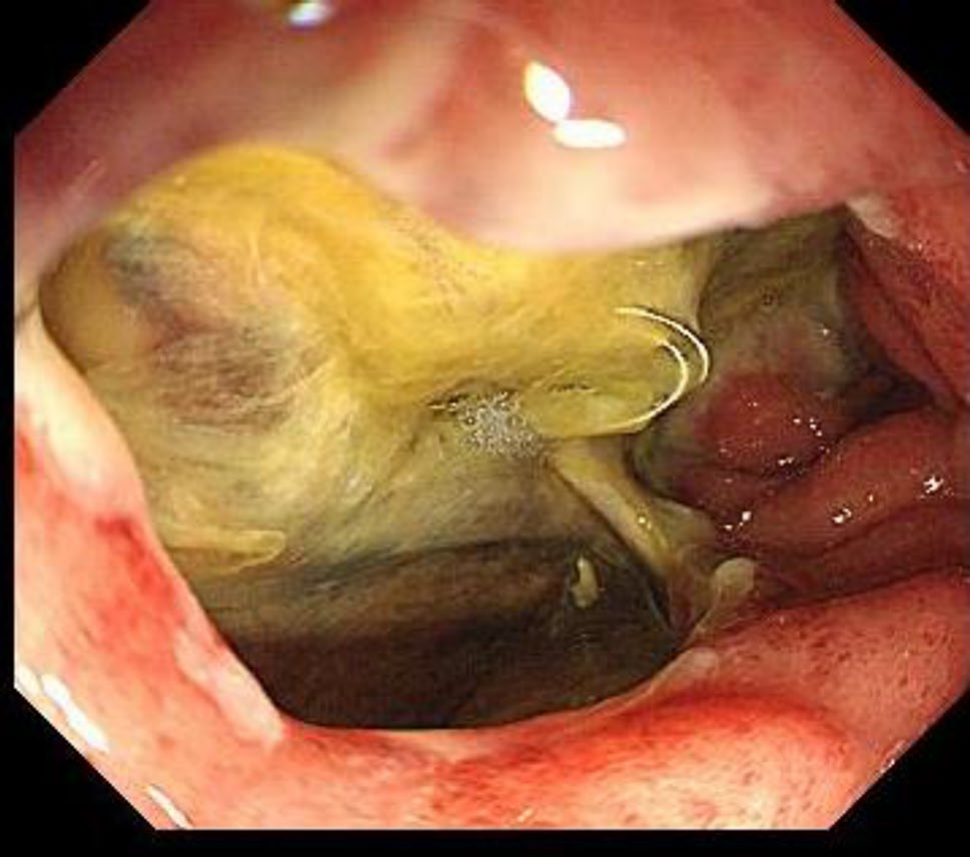
Deep irregular ulcer lesions extending from the duodenal bulb to the inner wall of the descending part

No adverse effects occurred, and thus, chemotherapy was shifted to an outpatient setting. However, the patient showed symptoms of bleeding approximately 1 month after discharge and was re-admitted. Upper gastrointestinal endoscopy showed a deep ulcer lesion that extended from the duodenal bulb to the inner wall of the descending part (Fig. 2). Penetration due to direct infiltration of the tumor was considered, but the bleeding site was not identified. The patient was treated accordingly and discharged. However, he was admitted again for severe re-bleeding. Abdominal angiography showed hemorrhaging in the area of the ulcer; thus, coil hemostasis was performed. However, the necrotic area became more extensive despite treatment, and he died of disseminated intravascular coagulation 1 week after angiography.

**Fig. 3:**
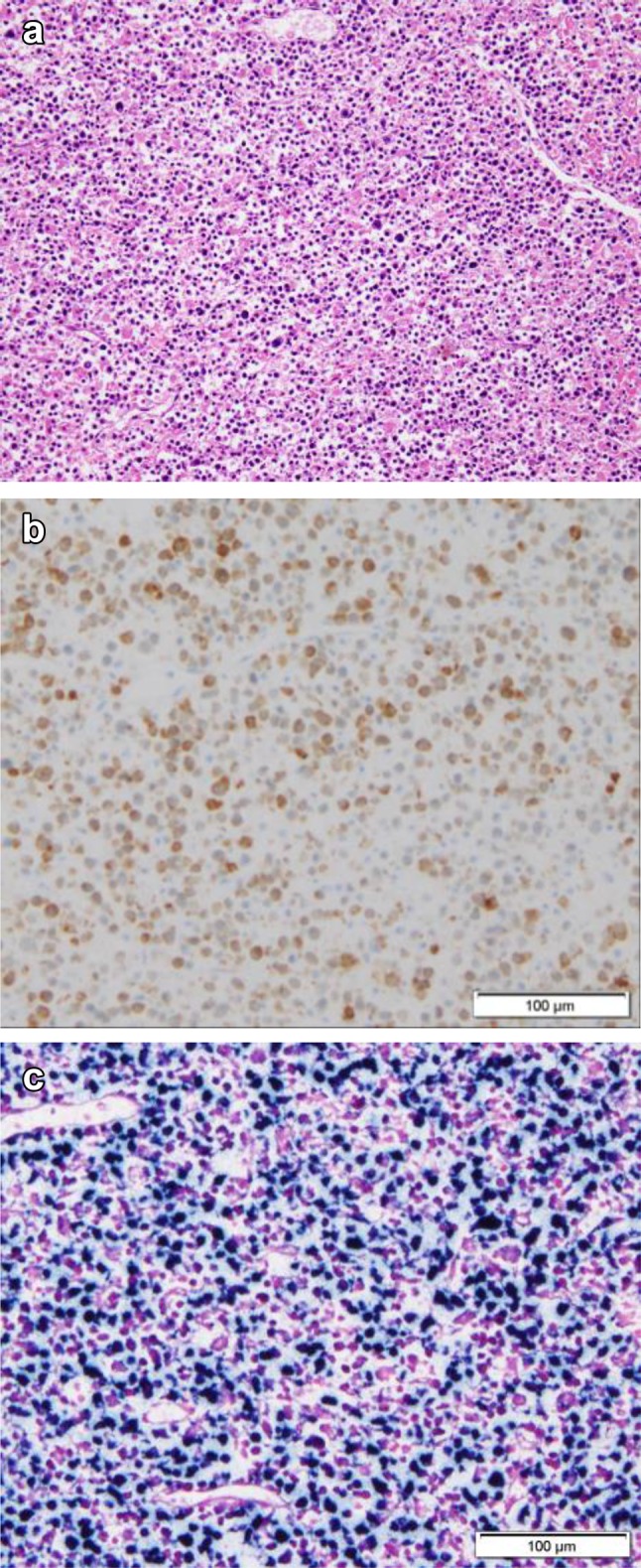
Tumor cells with medium to large and polymorphic bare nuclei showed proliferation with necrosis (**a**). The tumors were positive for CD3 (**b**) and on EBER-ISH (**c**)

Autopsy was performed after obtaining the family’s consent. We observed deep ulcerations extending from the duodenum to the head of the pancreas as well as diffuse white lesions that spread around the duct from the ulcer wall to the intrahepatic bile duct and gallbladder wall. Histologically, tumor cells with medium to large and polymorphic bare nuclear nuclei proliferated with the necrosis (Fig. 3a). Similar tumor cells were obtained from the spleen, lung, kidney, bone marrow, pancreas head, and adjacent lymph nodes, while arcuate cells were noted in the blood vessels. Diffuse proliferation was confirmed to be only present around the duct from the ulcer wall to the intrahepatic bile duct and gallbladder wall. Immunohistochemistry showed the following findings: CD3 (+) (Fig. 3b), CD5 (−), CD7 (+), CD20 (−), CD4 (−), CD8 (−), CD56 (+), TIA-1 (+), TdT, EBER-ISH (+) (Fig. 3c), AE 1/3 (−), MIB-1 (60–90%). Based on these findings, a pathological diagnosis of extranodal NK/T-cell lymphoma, nasal type, of the bile duct was made.

## Discussion

Malignant lymphoma is categorized as either lymphatic or extranodal (organ: primary site). Malignant lymphoma of the digestive system is rare and accounts for only 6–7% of the total cases of malignant lymphoma [1, 2]. The most frequently involved organ is the stomach (approximately 50% of the cases), followed by the small intestine (30–40%) and large intestine (approximately 10%). Lymphomas arising in the pancreatobiliary organs are rare; they are twice as common in men than in women. It usually develops in the pancreatic head and is localized [3, 4]. B-cell malignant lymphoma comprises the majority of extranodal lymphomas, while NK/T-cell lymphoma is rare, with only 10 cases that have been previously reported. The current case is only the eleventh case recorded [5–10].

For cases in which Epstein-Barr (EB) virus infection is considered to be involved in the development of extranodal NK/T-cell lymphoma, virus antibodies and EB viral DNA are necessary for diagnosis [11, 12]. Extranodal NK/T-cell lymphoma is generally a progressive cancer, except for those localized to the nasal cavity and the pharynx where it commonly originates. In the current case, no lesions were observed in the nasal cavity during both clinical assessments and autopsy.

Abdominal ultrasonography findings of malignant lymphoma of the pancreatobiliary organs show an irregular low echo mass in the pancreas, but this is nonspecific and difficult to differentiate from other pancreatic epithelial lesions. Meanwhile, it appears as a solid tumor image with poor contrast on abdominal CT and is characterized by poor scaling of the caudal pancreatic duct despite invasion of peripheral adipose tissue and lymph node enlargement. All these findings were observed in the current case [13].

Combination therapy with radiation therapy and chemotherapy has been attempted as treatment for NK/T-cell lymphoma, but it only yielded a median survival time of approximately 2 months even in treated cases, and the prognosis was extremely poor [14–16].

Histologically, extranodal NK/T-cell lymphomas show diffuse infiltration of tumor cells on HE staining. Other findings are as follows: (1) dissimilarity and polymorphisms are noted in tumor cells; (2) infiltration of inflammatory cells, such as lymphocytes, neutrophils, plasma cells, and macrophages, in the site; (3) granulation tissue with necrosis are noted in the tumor; and (4) Infiltration of the perivascular area or vascular destruction and angiocentric form.

Immunohistologically, extranodal NK/T-cell lymphoma cells show CD3 and CD56 positivity, while CD4, CD8, and T-cell-related antigens are negatively expressed. Regarding the expression of EB virus-related antigens, NK cell surface antigen (CD56) and EB virus-encoded RNAs (EBER) show positive results on in situ hybridization (ISH). In the current case, immunohistochemistry showed CD3 (+), CD56 (+), CD4 (−), CD8 (−), TdT (−) while EBER-ISH also tested positive; these findings were consistent with the histological form of this disease [17–19].

In conclusion, in cases of pancreatic tumors where tissue diagnosis via biopsy cannot be performed despite indications of a malignancy, tissue diagnosis needs to be performed more carefully while considering the possibility of lymphoma. Immunostaining and EB virus tests should also be performed. NK/T-cell lymphoma shows rapid tumor progression, and it may cause sudden patient deterioration due to gastrointestinal infiltration. Thus, NK/T-cell lymphoma should be ruled out in patients diagnosed with pancreatic tumors.
